# Clinical measurement and categorization of optic disc in glaucoma patients

**DOI:** 10.4103/0301-4738.55075

**Published:** 2009

**Authors:** Harsha B L Rao, G C Sekhar, Ganesh J Babu, Rajul S Parikh

**Affiliations:** VST Glaucoma Centre, Kallam Anji Reddy Campus, L V Prasad Eye Institute, L V Prasad Marg, Banjara Hills, Hyderabad, India

**Keywords:** Heidelberg retinal tomograph

## Abstract

**Background:**

Assessment of optic disc size is an important component of optic nerve head examination. Agreement between different methods of disc size measurements is not very good.

**Purpose:**

To assess the agreement between the disc size assessed by Heidelberg retina tomograph (HRT) and stereobiomicroscopy with a 90 diopter (D) lens. To report the clinical (measured by biomicroscopy) disc diameters of small, average and large optic discs categorized by HRT disc areas.

**Setting and Design:**

Observational study of subjects examined in the glaucoma clinic of a tertiary eye institute.

**Materials and Methods:**

Seventy-five eyes of 75 glaucoma subjects were studied. Disc diameter was measured using stereobiomicroscopy and HRT. The agreement between the two sets of measurements was assessed by intraclass correlation coefficient (ICC). Discs were classified into small (<1.6 mm^2^), average (1.6-2.6 mm^2^) and large (>2.6 mm^2^) depending on cutoffs provided by the manufacturers of HRT. The means (95% CI) of the corresponding vertical disc diameter in these groups were assessed.

**Statistical Analysis:**

ICC, Bland and Altman plots.

**Results:**

ICC for measurements of clinical and HRT horizontal disc diameter was 0.518 and for vertical disc diameter measurement was 0.487. The mean difference between the clinical and HRT measurements as analyzed by the Bland and Altman plot was 0.17 (95% CI, 0.13- 0.47) for horizontal and 0.22 (95% CI, 0.11- 0.54) for vertical disc diameter. Of the 75 eyes, 3 eyes had small discs, 54 average and 18 large discs. The mean clinical vertical disc diameter for small discs was 1.55 mm (95% CI, 1.2-1.7), for average discs was 1.91 mm (95% CI, 1.87-1.96) and for large discs was 2.15 mm (95% CI, 2.03–2.27).

**Conclusion:**

The agreement between clinical and HRT disc diameter measurements is moderate. Disc diameter measurement on stereobiomicroscopy can be used to categorize discs into small, average and large discs.

Assessment of optic disc size is an important component of optic nerve head examination, as optic disc parameters such as neuroretinal rim area and cup-disc ratio vary with the disc size. The Blue Mountain study has shown that cup-disc ratio is strongly associated with disc diameter and optic discs with larger vertical diameters have considerably greater vertical cup-disc ratios.[1] Correction for disc size is necessary for optic disc variables when ranking these variables for detection of glaucomatous optic nerve damage.[2] It is debatable whether disc size is an independent risk factor for glaucoma.[3] Disc size is known to vary largely between populations, among individuals and between eyes.[4,5] Differences in the techniques of measuring disc size across studies could result in different estimates of disc size, making comparisons among studies[6,7] difficult. These differences must be considered while evaluating the optic disc. In practice it is not necessary to actually measure the disc size but is enough to classify it as large, medium or small disc on clinical examination.[8] We evaluated the disc diameter in the clinic by slit lamp biomicroscopy with a +90 diopter (D) lens and looked at its agreement with measurements taken using confocal scanning laser ophthalmoscopy. The aim of the study was to correlate the disc diameter assessed by biomicroscopy of small, average and large discs as categorization by Heidelberg retina tomography (HRT).

## Materials and Methods

This is an observational study of 75 eyes of 75 non-consecutive subjects attending the glaucoma clinic at our institute between January 2007 and March 2007. The ethics committee of the L.V. Prasad Eye Institute, Hyderabad approved the study protocol. The methods applied in the study adhered to the tenets of the declaration of Helsinki for the use of human subjects in biomedical research.

Inclusion criteria were age above 18 years, best corrected visual acuity of 20/40 or better, good images on HRT as defined by interscan standard deviation (SD) of ≤50 μm and willingness to participate in the study. The exclusion criteria were refractive errors exceeding 5.0 D sphere and/or 3.0 D cylinder, any media opacity precluding imaging techniques or clinical examination of the disc and inability to undergo the tests.

All subjects underwent a comprehensive ophthalmic examination. The vertical and horizontal disc diameters were recorded after mydriasis, at the slit lamp with a +90 D double-aspheric fundus lens (Volk Opticals, Mentor, OH), by a single observer (HBL). A narrow slit beam of the Haag-Streit slit lamp (Haag-Streit BM 900® V, Haag-Streit AG, Koeniz, Switzerland), with its width maintained constant, was progressively reduced in size from 8 mm until it coincided with the diameter of the disc. The beam length was then recorded from the millimeter scale of the instrument. Because the slit lamp beam length is calibrated to 0.1 mm, the reading was approximated to the nearest 0.1 mm. After each reading, the millimeter scale was reset to 8 mm. The length of the beam was adjusted first to the vertical and then to the horizontal diameter of the disc and read on the scale of the slit-lamp in millimeters. The measurement was taken only if the optic disc was in good focus and was seen in the center of the image field. This reading was then multiplied with a correction factor of 1.41 to obtain the actual disc diameter. Direct measurements from the slit lamp of small, average and large disc groups are also reported.

The intra-and inter-observer agreements for disc diameter measurements were studied in the initial 40 subjects who fulfilled the inclusion and the exclusion criteria. These subjects were not included in the study cohort of 75 subjects. Intra-observer agreement was assessed as follows. The observer (HBL) aligned the height of the slit beam to the diameter of the disc and the reading on the slit lamp scale was noted by an assistant. The observer was masked to the reading. After measuring the vertical and horizontal disc diameters of both the eyes, the slit beam was opened to full height and the disc diameters of the first eye was once again measured by aligning the height of the slit beam to the diameter of the disc and the reading on the slit lamp scale was noted by a second assistant who was masked to the reading noted down by the first assistant. The observer (HBL) was masked to both these measurements. Inter-observer agreement was assessed between the first set of readings of the first observer (HBL) and the disc diameter measurements obtained by an experienced observer (GCS) on the same 40 subjects, to validate the measurements of the first observer.

HRT-II (Heidelberg Engineering, GmbH, Dossenheim, Germany) examination was performed after entering the subject's manifest refraction. Three scans were acquired automatically after initial positioning by the operator. All scans were assessed subjectively for the presence of good optic nerve head (ONH) centration, focus and uniform illumination. All scans had interscan standard deviation of < 50 *μ*m. Scans with poor image quality were excluded. An experienced operator (GJB), masked to the clinical measurements, marked the optic disc margin as the inner border of the Elschnig ring. Contour lines were placed on the margin of the optic disc according to the instructions provided on the HRT Web site (http://www.heidelbergengineering.com). HRT disc area was recorded. Vertical and horizontal disc diameters were calculated using the interactive measurements option.

One randomly selected eye of each subject was chosen for analysis. The agreement between clinical and HRT disc diameter measurements was analyzed by intraclass correlation coefficient (ICC) and the Bland and Altman method. SPSS version 13 and Medcalc version 9 were used for statistical analyses.

Based on the standard deviation of a mean difference of 0.5, to estimate an upper agreement limit with an absolute error of 0.2 at 95% confidence level, 73 subjects were required.

The discs were then classified into small, average and large depending on the disc area cut-offs provided by the manufacturers of HRT.[9] Discs with area less than 1.6 mm^2^ were classified as small; 1.6-2.6 mm^2^ as average and more than 2.6 mm^2^ as large. Mean and 95% confidence limits of the disc diameter by 90D examination- both with and without magnification factor correction- were determined in the corresponding groups.

## Results

The characteristic features of the 75 subjects are shown in Table 1. All the subjects were of Indian origin. Break-up according to the diagnosis of these 75 subjects is shown in Table 2. ICC for intra-observer agreement for horizontal disc diameter was 0.971 and for the vertical disc diameter was 0.937. The ICC for inter-observer agreement for horizontal disc diameter was 0.889 and for vertical disc diameter was 0.912. ICC for agreement between clinical and HRT horizontal disc diameter was 0.518 and for vertical disc diameter was 0.487.

**Table 1:** Characteristic features of the study cohort

Age (Mean, SD)	52.5 years (12.9)
Gender (Male:Female)	47(62.7%): 28(37.3%)
Best Corrected Visual Acuity (Mean, SD)	0.03 (0.08)
Spherical Equivalent (Mean, SD)	0.1 (1.9)
Systemic Diseases	
Diabetes n(%)	19 (25.3%)
Hypertension n(%)	25 (33.3%)

**Table 2:** Break up of subjects according to the diagnosis

Diagnosis	Number (%)
Disc suspect	25 (33.3)
Primary open angle glaucoma	21 (28)
Angle closure disease	16 (21.3)
Normal tension glaucoma	9 (12)
Juvenile open angle glaucoma	2 (2.7)
Ocular hypertension	1 (1.3)
Others	1 (1.3)

**Figure 1:**
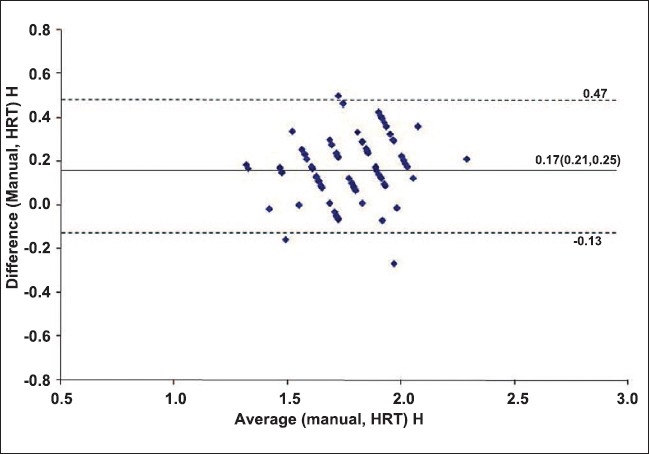
Bland and Altman plot for agreement between clinical and HRT horizontal disc diameters

**Figure 2:**
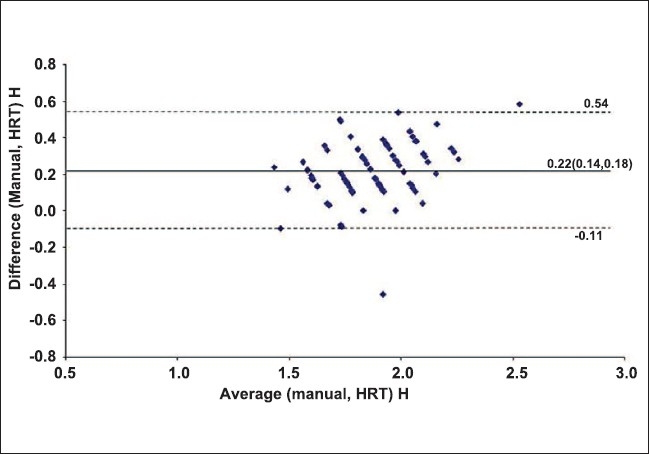
Bland and Altman plot for agreement between clinical and HRT vertical disc diameters

The agreement between the clinical and HRT measurements of disc diameter was analyzed by the Bland and Altman method [Fig 1 and 2]. The mean difference was 0.17 (95% CI, 0.13- 0.47) for horizontal disc diameter and 0.22 (95% CI, 0.11- 0.54) for vertical diameter.

The mean (95% CI) vertical disc diameter of the three groups based on the disc size is shown in Table 3. It was 1.55 (1.2-1.9) in the small disc group, 1.91 (1.87-1.96) in the average disc group, and 2.15 (2.03-2.27) in the large disc group. When the direct measurement from the slit lamp was analyzed without correction for the magnification factor, the mean (95% CI) vertical disc diameter was 1.1 (0.85, 1.34) in the small disc group, 1.36 (1.32, 1.39) in the average disc group and 1.52 (1.44, 1.61) in the large disc group.

**Table 3:** Mean and 95% CI of HRT and clinical vertical disc diameters in small, average and large discs

Category	Number	Mean HRT vertical disc diameter (95% CI)	Mean clinical vertical diameter (95%CI)
Small (<1.6 mm^2^)	3	1.4 (1.04, 1.76)	1.55 (1.2, 1.9)
Average (1.6 – 2.6 mm^2^)	54	1.71 (1.67, 1.75)	1.91 (1.87, 1.96)
Large (>2.6 mm^2^)	18	1.95 (1.88, 2.02)	2.15 (2.03, 2.27)
Total	75		

## Discussion

It has been shown by many studies that measurements of ONH parameters are confounded by the optic disc size.[1–3] To address this issue in a clinical perspective, it is not necessary to accurately measure the disc size but it is enough to categorize discs into small, average and large discs.[8] HRT measurements of the optic disc size are well documented for different ethnic groups. Since the access to such costly instruments is restricted; it is useful to have a clinical means of categorizing the disc by its size. Measuring the disc diameter on slit lamp biomicroscopy with a high-powered convex lens is a good alternative. In this study we report the validation and potential use of this method.

Good intra and inter observer agreement are prime requirements for any diagnostic test. The ICC for intra and interobserver agreement for vertical disc diameter measurement was 0.937 and 0.912 respectively. This is in agreement with the intra- and interobserver variability of optic measurements by slit lamp biomicroscopy.[10,11] The other advantages of this technique for determining the optic disc size is easy accessibility compared to other expensive imaging techniques.

HRT was chosen for comparison in this study as the optic disc measurement with HRT correlated well with the measurements from histological studies,[12] *in vivo* measurements of optic disc diameter performed during vitrectomy[13] and also the good reproducibility reported with HRT.[14]

There have been studies looking at the agreement between fundoscopy and HRT for cup-disc ratio,[15,16] but those for the disc diameter are limited.

Lim *et al*. compared the vertical disc diameters obtained with 60, 78 and 90 D lenses with that of HRT.[17] They found that the correlation was moderate to good between the two (r=0.80 with 60D lens and r=0.59 with 90D lens). In their study, optic disc size measured with HRT was larger than that with 90D lens. In our study, disc diameter measurement with HRT was less than that obtained with clinical method. Instead of correlation, we calculated ICC because correlation gives the strength of relationship between the methods while ICC gives the actual agreement between the methods.[18]

Barkana *et al*.[19] assessed the interchangebility of optic disc diameter measurements with slit-lamp biomicroscopy with a 78D lens and HRT 2. The mean difference between HRT and 78D lens measurements was 0.12 mm and the limits of agreement between these methods were poor (-0.29 to 0.53 for HRT and slit-lamp microscopy). In our study, the mean difference between clinical and HRT measurements as analysed on Bland and Altman plots was 0.17 (95% CI, 0.13- 0.47) for horizontal disc diameter and 0.22 (95% CI, 0.11-0.54) for vertical diameter. In Barkana, *et al*.'s study too, disc diameter measurement with HRT was greater than the clinical measurement. Barkana *et al*. also evaluated the agreement between these methods for classification of discs into small, average and large disc categories and this was again less than moderate (Kappa statistics κ = 0.154, 95% CI 0-0.39). They concluded that HRT and clinical disc measurement methods cannot be used interchangeably in clinical practice and research.

Disc diameter measurement with HRT was always lesser than that by clinical method in our study which is contrary to that found by the above two studies. We cannot explain this but it might be due to the operator dependent subjectivity involved in delineating the disc margin in HRT.

Jonas recommended that in clinical practice, it is enough to document the disc size as average, small or large.[8] There is no validated method to classify discs into small, average and large categories. We have used the classification proposed by manufacturers of HRT.[9] These measurements would help the clinician to classify optic nerve heads into small, medium and large on routine clinical evaluation without dependence on expensive technology. It is ideal to have the cup-disc ratio corrected for the disc size but short of that, the above classification can help one corroborate with other clinical signs and decide if a 0.3:1 cup is pathological in a small disc or if a 0.7:1 cup is physiological in a large disc. We tried to group discs into these categories based on the HRT data and tried to provide the corresponding clinical disc diameter measurements, which would benefit those not using HRT. We have also provided the direct measurements from the slit lamp without magnification correction for the lens so that those using a 90 D lens can directly classify the discs into small, average or large without performing any complex calculations.

One of the limitations of the study is the use of a non-consecutive sample. As the study was basically about determining the agreement between two methods and did not involve summarizing disease characteristics, a non-consecutive sample would not have affected the results to a great extent.

This study is also limited by the number of small and large discs, which is reflected in the overlapping confidence intervals of the groups. While the sample size is adequate for agreement study, it is not adequate for classification of disc size into groups. Larger sample size with tighter confidence intervals is needed for this.

In conclusion, our study is in agreement with the previous studies. The agreement between measurements by HRT and the clinical method is only moderate. Disc diameter measurement on stereobiomicroscopy can be used to categorize discs into small, average and large discs.
